# Role of mineral nutrients, antioxidants, osmotic adjustment and PSII stability in salt tolerance of contrasting wheat genotypes

**DOI:** 10.1038/s41598-022-16922-9

**Published:** 2022-07-25

**Authors:** Nadeem Hussain, Younas Sohail, Nasir Shakeel, Muhammad Javed, Hussan Bano, Hafiza Saima Gul, Zafar Ullah Zafar, Islam Frahat Zaky Hassan, Abdul Ghaffar, Habib-ur-Rehman Athar, Rahaf Ajaj

**Affiliations:** 1 Institute of Pure and Applied Biology, Bahauddin Zakariya University, Multan, 60800 Pakistan; 2 Department of Botany, Emerson University, Multan, Pakistan; 3 Department of Inorganic, Analytical Chemistry and Electrochemistry, Faculty of Chemistry, Silesian University of Technology, Gliwice, Poland; 4 Department of Botany, Division of Science and Technology, University of Education, Lahore, Pakistan; 5 Department of Botany, The Women University, Multan, Pakistan; 6 Water Relations and Field Irrigation Department, Agricultural and Biology Research Institute, National Research Center, Cairo, Egypt; 7 College of Health Sciences, Abu Dhabi University, Abu Dhabi, UAE

**Keywords:** Plant sciences, Plant physiology, Plant stress responses

## Abstract

Global food production is threatened due to increasing salinity and can be stabilized by improving salt tolerance of crops. In the current study, salt tolerance potential of 40 local wheat cultivars against 150 mM NaCl stress was explored. Salt treatment at seedling stage caused less reduction in biomass, K^+^ and P while more decline of Na^+^ in tolerant cultivars due to reduced translocation and enhanced exclusion of Na^+^ from leaves. Principal component analysis based selected S-24, LU-26S, Pasban-90 (salt tolerant) and MH-97, Kohistan-97, Inqilab-91 and Iqbal-2000 (salt sensitive) cultivars were evaluated at adult stage applying 150 mM salinity. Osmotic adjustment by accumulation of soluble sugars and proline and accelerated antioxidant enzymes activities caused efficient scavenging of reactive oxygen species making S-24 and LU-26S tolerant while in MH-97 and Kohistan-97, high MDA represent greater membrane damage due to oxidative stress making them salt sensitive. Chlorophyll *a* fluorescence transients confirmed better efficiency of photosystem II in S-24 and LU-26S based on energy fluxes (ABS/RC, TRo/RC, ETo/RC and DIo/RC), performance index (PI_ABS_) and maximum quantum yield (Fv/Fm). These findings can be correlated using molecular techniques to identify genes for salt exclusion, osmotic adjustment and photosynthetic activity for use in molecular breeding programs.

## Introduction

Soil salinity is a major abiotic stress causing crop losses up to $ 31 million. According to estimates, 45 million hectares (Mha) out of 230 Mha irrigated land of the world is salt affected^1^. Osmotic effect due to Na^+^ in soil causes stomatal closure, reduces transpiration, disturbs plant water status and inhibits leaf expansion^2^, while specific ion effect reduces plant’s ability to uptake other ions such as Ca^2+^, K^+^ and Mg^2+^ affecting distribution of essential nutrients in plants^3^ resulting premature senescence of leaves, yield reduction and plant death^4^.

Decreasing salt transport to shoots, increasing intracellular sequestration of salts into vacuoles of leaves, oxidative stress tolerance and osmotic adjustment are important strategies used by plants for salt tolerance^2,5^. Overproduction of ROS in chloroplast during oxidative stress causes photo-damage reducing the efficiency of photosynthetic electron transport as well as lipid peroxidation of plasma membrane leading to production of MDA which is an indicator of damage caused by salt stress^6^. To mitigate the toxic effects of ROS, plants have various enzymatic such as ascorbate peroxidase (APX), superoxide dismutase (SOD), peroxidase (POD), catalase (CAT), glutathione peroxidase (GPX) and non-enzymatic (tocopherol, ascorbate, glutathione, carotenoids) antioxidant systems^7^ . Photosynthetic machinery is affected by salinity but the mechanism of decreasing photosynthesis is complex and unclear^8,9^. Data from raw chlorophyll *a* fluorescence transients (OJIP) was used to calculate JIP-test parameters, such as the maximal energy fluxes for absorption (ABS/RC), trapping (TRo/RC), electron transport rate (ETo/RC) and energy dissipation (DIo/RC) per reaction centre^6^, reduction of end electron acceptors (RE), maximum quantum yield of PSII (Fv/Fm) and performance index on absorption basis (PI_ABS_)^10^ that explain structure and function of PSII and the possible flow of electron in thylakoid membranes. O, J, I, P are different steps of polyphasic fluorescence transients in which O refers to minimal fluorescence (Fo), ‘J’ and ‘I’ are intermediate steps which represent fluorescences Fj and Fi respectively while ‘P’ refers to maximum fluorescence (Fm). Salinity mediated osmotic stress triggers the inactivation of PSII reaction centre reducing its quantum yield.

Tremendous genetic variations for salinity tolerance in wheat gene pool exist and some wheat genotypes have been developed in previous years showing greater salt tolerance^11^, a large knowledge gap still exists to understand genetic and physiological basis of salt tolerance in new local and exotic germplasm collection and its application in introgression salinity tolerance in wheat^12,13^. So it is suggested that salt tolerance in wheat can be improved by selective breeding using physiological and biochemical selection criteria, which play important role in sustainable management of salt affected soils^11,14^. It is hypothesized that a novel source of salt tolerance is present in new local wheat germplasm collection of wheat, which can be used for developing salt tolerant wheat cultivars through selective breeding. However, pre-breeding require a detailed characterization of germplasm. The present study was aimed to assess genotypic variability in physiological and biochemical traits in new local germplasm collection of wheat, particularly those are related to photosystem-II activity. The secondary objective of the study was to draw the relationship between studied traits and degree of salt tolerance.

## Materials and methods

Experimental research and field studies on plants including the collection of plant material complies with relevant institutional, national and international guidelines and legislation.

Present research study consisted of three experimental parts:

### Screening of germplasm against salt stress at seedling stage

Germplasm of 40 locally grown wheat cultivars, obtained from Ayyub Agricultural Research Institute (AARI), Faisalabad, Arid Zone Research Institute (AZRI), Bhakkar and Regional Agricultural Research Institute (RARI) Bahawalpur, Pakistan was screened out at seedling stage in wire-net house of the Botanic Gardens of Bahauddin Zakariya University, Multan (30° N and 71° 28 E) Pakistan. The seeds after surface sterilizing with 5% sodium hypochlorite solution for five minutes to avoid any fungal infection and washing thoroughly with distilled water were sown in plastic bowls of 24 × 24 × 10 cm (length × width × depth) size obtained from G.M. Scientific Store, Multan, Pakistan. A total of 40 bowls were taken and divided into two groups of 20 bowls each. The growth medium used was well washed river sand (3 kg in each bowl). Seeds of two varieties (10 of each) were sown in each bowl in two separate rows. One group of plants (control) was given full strength Hoagland’s nutrient solution^15^, and second group was given 150 mM NaCl solution in full strength Hoagland’s nutrient solution. To maintain salinity stress level and compensate evapo-transpirational water loss, 100 mL distilled water was supplied. In addition, nutrient solutions were replaced weekly to replenish nutrient status in their growth medium. After 21 days of the experiment, the plants were harvested, washed thoroughly with distilled water. Fresh weight of shoot and root of the harvested plants were measured in electric balance (BSM-220.4, Hanchen, China) in milligrams (mg) immediately after carefully separating the root from shoot. The separated root and shoot of plants were dried in an electric oven (UN-110, Memmert, Germany) at 75 °C for 72 h after putting in a Kraft paper bag and their dry biomass (mg) was taken.

The dried plant samples were ground into fine powder and digestion mixture (Li_2_SO_4_.2H_2_O, Se, H_2_O_2_ and conc. H_2_SO_4_) was used to digest plant sample (0.2 g) at 350 °C on a hot plate, diluted to 50 mL and used to determine Na^+^ and K^+^ by flame photometer (Jenway, PFP-7, UK) as described by Allen, et al.^16^. Jackson^17^ method was used to measure P (1 mL filtrate, 4 mL distilled water and 550 µL Bartons reagent) noting absorption at 470 nm by spectrophotometer (Jenway, 6850, UK).

### Appraisal of morpho-physiological and biochemical attributes of selected cultivars at adult stage

The selected (three salt tolerant and four sensitive) cultivars were sown in plastic pots (30 × 30 cm; diameter and height) filled with 8 kg of well washed river sand and arranged in two groups (35 of each), one for control (non-saline) and other for saline (150 mM NaCl) solution. The pots were filled with 2 L of full strength Hoagland’s nutrient solution and eight seeds of each variety were sown in a pot. The experimental lay out was completely randomized design (CRD) with five replicates under normal environmental conditions. After germination, thinning was performed and four healthy, equidistant plants were left in each pot. Salt stress (50 mM NaCl in Hoagland’s nutrient solution) was applied to one group of plants after 21st day of germination, increased to 100 mM and finally to 150 mM in alternating days to avoid sudden osmotic shock, the control plants received only Hoagland’s nutrient solution in tap water. Final salt treatment level was maintained till the end of experiment. After 60 days of sowing two plants were harvested from each pot, their root and shoot were separated and fresh and dry biomass were measured on electric balance. Dried samples were ground into fine powder to quantify mineral elements (NPK and Na^+^). Different parameters including quantum yield of PSII, chlorophyll contents (SPAD values), leaf water relations, proline, total carbohydrates, soluble sugars, starch, soluble proteins, amino acids, MDA and anti-oxidant enzymes were measured. The experiment proceeded with two plants per pot, finally the plant height and flag leaf area were measured, the plants were harvested and their yield attributes were determined. Analysis of the data collected from these parameters recommended two most salt tolerant (S-24 and LU-26S) and two most salt sensitive sensitive (MH-97 and Kohistan-97) cultivars. Only organic osmolytes, chlorophyll content, MDA and anti-oxidant enzymes have been discussed in this manuscript.

### Measurements

#### Leaf proline

Proline was measured according to Bates, et al.^18^ methodology in which 0.25 g fresh leaf sample ground in 3% sulpho-salicylic acid in a chilled pestle and mortar was vortexed by adding acid ninhydrin solution and toluene. Absorbance of upper layer (chromophore) was recorded by spectrophotometer at 520 nm and concentration of proline was measured with the help of a standard curve.

#### Total carbohydrates, total soluble sugars, starch and chlorophyll content

Total soluble sugars and starch were measured according to Yemm and Willis^19^ by adding 80% ethyl alcohol in 0.2 g dried leaf sample, while for determining total carbohydrates 6 N HCl was added^20^ vortexed overnight and diluted to 50 mL. Total carbohydrates, starch and total soluble sugars were estimated after heating the filtrate in anthrone solution and recording absorbance at 625 nm by UV spectrophotometer. Chlorophyll content (SPAD index) was estimated by a portable chlorophyll meter (Minolta, Chlorophyll meter, SPAD-502, Japan). Average of three readings from each flag leaf was taken.

#### Lipid peroxidation

Extant of leaf lipid peroxidation can be estimated as MDA content by Cakmak and Horst^21^ method. In leaf sample ground in 0.1% trichloro-acetic acid (TCA), added reaction solution (2.5 g thiobarbituric acid (TBA) in 5% TCA solution) and heated, absorbance at 532 and 600 nm was noted after cooling the solution.

#### Antioxidant enzymes assay

0.4 g plant sample ground in 4 mL sodium phosphate buffer (50 mM, pH 7.8) in a chilled pestle and mortar was homogenized and centrifuged at 6000–8000 × *g* for 20 min and supernatant was used for measuring antioxidant enzymes activity.

SOD (EC 1.15.1.1), activity was assayed following the method of Giannopolitis and Ries^22^. Reaction solution consisting of nitroblue tetrazolium (NBT), methionine, riboflavin, Ethylene-diamine tetra-acetic acid (EDTA), phosphate buffer, distilled water and enzyme extract were placed under 30 W (W) fluorescent lamps for 10 min. Tubes without enzyme extract were taken as control, while the blank solution contains reaction solution including enzyme extract placed in dark. Absorbance was noted at 560 nm with UV–visible spectrophotometer. APX (EC 1.11.1.11), concentration was calculated according to Nakano and Asada^23^ with some modifications. Reaction solution contains sodium phosphate buffer, enzyme extract, ascorbic acid and H_2_O_2_. Absorbance was recorded after 0, 30 and 60 s interval at 290 nm by spectrophotometer and mean OD was taken. The method described by Aebi^24^ was used to measure CAT (EC 1.11.1.6) activity from solution containing enzyme extract, sodium phosphate buffer and H_2_O_2_ and absorbance was taken at 240 nm at 0, 30 and 60 s interval. POD (EC 1.11.1.7) activity was assayed by taking enzyme extract, sodium phosphate buffer, H_2_O_2_ and guaicol. Absorbance at 0, 30 and 60 s interval was measured at 470 nm by spectrophotometer according to Change and Maehly^25^.

### Evaluation of PSII response of selected cultivars against salt stress at vegetative stage

Analysis conducted on the basis of data obtained from different parameters at adult stage experimentation allowed us to screen two most salt tolerant (S-24 and LU-26S) and two most salt sensitive cultivars (Kohistan-97 and MH-97) which were further grown in plastic pots like adult experiment with control and salinity treatment blocks having five replicates of each variety and CRD experimental lay out. On the basis of chlorophyll, *a* fluorescence transient’s measurements by JIP-test, tolerant and sensitive nature of selected cultivars have been confirmed.

#### Fast chlorophyll a fluorescence transient (OJIP)

Middle part of the 3rd mature leaf of each plant was used to measure fluorescence parameters, 40 min after sunset to ensure dark adaptation of leaves and then exposed to strong actinic light (3000 μmol m^−2^ s^−1^) with hand held chlorophyll fluorescence metre (FluorPen FP-100 MX-LM, Photon System Instruments, Czech Republic) from 20 μs to 2 s. The data was recorded on the basis of literature available on websites of chlorophyll fluorescence meters manufacturers using computer software FlourPen v 1.0.4.0. Structural and functional changes in PSII under salinity stress were estimated on the basis of various biophysical and phenomenological energy fluxes from fluorescence data such as: Fo = Minimum fluorescence, Fj = Fluorescence intensity at J phase of OJIP, Fi = Fluorescence intensity at I phase of OJIP, Fm = Maximum fluorescence, Fv = Variable fluorescence, Vj = Variable fluorescence at phase J of fluorescence transient curve, Vi = Variable fluorescence at phase I of fluorescence transient curve, Fm/Fo = Electron transport rate through PSII, Fv/Fo = Ratio of photochemical to non- photochemical quantum efficiency, Fv/Fm = Maximum quantum yield of primary photochemistry, Mo = Maximum rate of accumulation of closed reaction centres, Area = Area above the fluorescence between Fo and Fm, Phi-Po = Quantum yield for primary photochemistry, Psi-o = Capacity of PS II to transfer trapped excitation, Phi-Eo = Quantum yield of electron transported beyond QA, Phi-Do = Maximum quantum yield of non-photochemical de-excitation, Phi-Pav = Average performance index, Pi-Abs = Photosynthetic performance index on absorption basis, ABS/RC, TRo/RC, ETo/RC and DIo/RC = Absorption, Trapped energy flux, Electron transport and Dissipation energy flux per reaction centre.

### Statistical analysis

MS Excel software was used for graphical representation of the data. Screening at seedling stage was done by PCA which is a multivariate data analysis technique conducted by using Origin 2019 software (OriginLab, Northampton, MA, USA). The adult experimental plan was CRD with five replications. Data was analyzed using the Costat program (Version 6.303, USA). Means ± SE were compared with least significant difference (LSD) test at 5% level of significance by Snedecor and Cochran^26^.

### Ethical statement

It is certified that preparation of this manuscript is strictly in compliance with the ethical standards section of the journal.

### Consent for publication

All authors agree to submit this manuscript in “*Scientific Reports*”.

## Results

### Screening of germplasm

**Figure 1:**
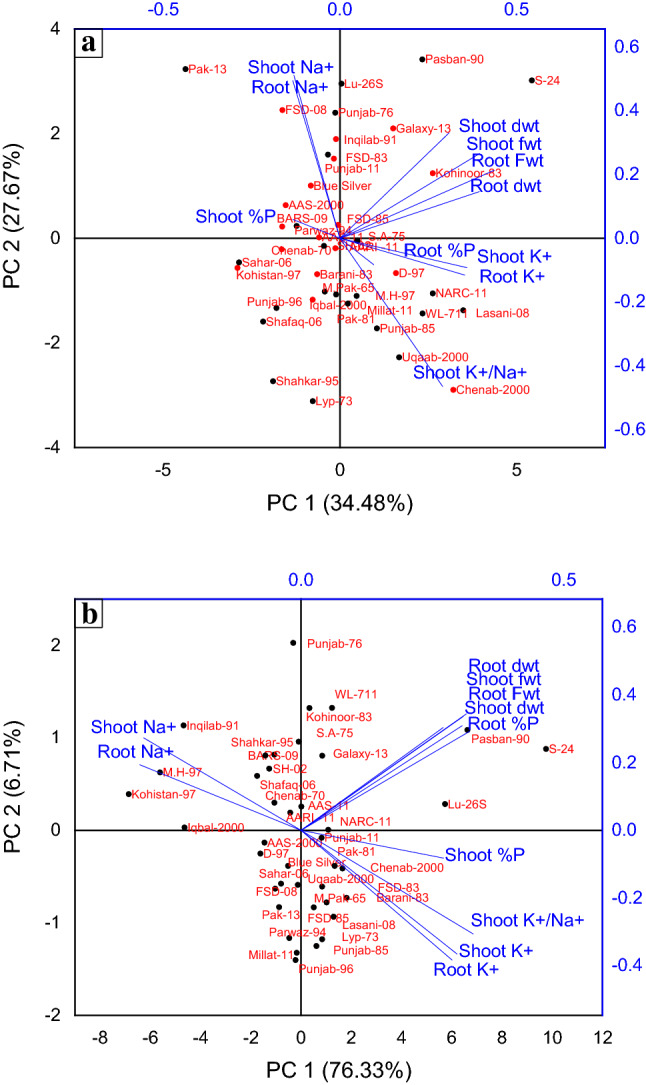
Biplots of PCA for different parameters to screen 40 wheat cultivars at seedling stage under (**a**) Control conditions (**b**) Saline conditions.

All the cultivars grown in saline medium (150 mM NaCl) showed reduction in biomass, K^+^ and P concentration of shoot and root while increase in Na^+^ concentration. PCA was performed on the data obtained from fresh and dry biomass, Na^+^, K^+^, K^+^/Na^+^ ratio and P concentration of shoot and root of 40 local wheat cultivars at seedling stage. The different variables studied have been transformed into different factors with first factor contributing most variability and last factor contributing least variability. Eigenvalue is equal to total variance and sum of all eigenvalues is equal to number of variables studied. Original variable scores have been transformed into principal components. The axis or vector with major contribution is called principal component 1 and subsequent orthogonal axes as PC2, PC3 and so on. The Eigen values of the first four components and their variability percentage assessed from scree plot (Figure of scree-plot not given) for control and salinity stressed plants are presented in Table 1. PCA reduced the eleven parameters to four components in control plants with 62.15% of the total variations in which first component (PC1) accounts for 34.48% and second component (PC2) for 27.67% while for salinity stressed plants, they accounted for 76.33% and 6.71% respectively with 83.04% of the total variations (Fig. 1a, b).

**Table 1:** The Eigen values of the first four components and their variability percentage under control and saline conditions.

Treatment	Principal component	Eigen value	% age of variance (%)	Cumulative (%)
Control	1	3.79304	34.48	34.48
	2	3.04422	27.67	62.16
	3	1.57485	14.32	76.47
	4	0.97995	8.91	85.38
Saline	1	8.3959	76.33	76.33
	2	0.7377	6.71	83.03
	3	0.67833	6.17	89.20
	4	0.41331	3.76	92.96

The bi-plot of PCA showed almost random distribution of genotypes under control conditions (Fig. 1a) however it showed specified grouping under saline conditions clearly separating tolerant and sensitive genotypes (Fig. 1b). Analysis conducted on the basis of data obtained from different parameters at seedling stage allowed us to screen three most salt tolerant (S-24, LU-26S and Pasban-90) and four most salt sensitive cultivars (Kohistan-97, MH-97, Inqilab-91 and Iqbal-2000).

### Adult stage experiment

The cultivars selected from screening experiment, were grown and different parameters measured at adult stage have following results.

**Figure 2:**
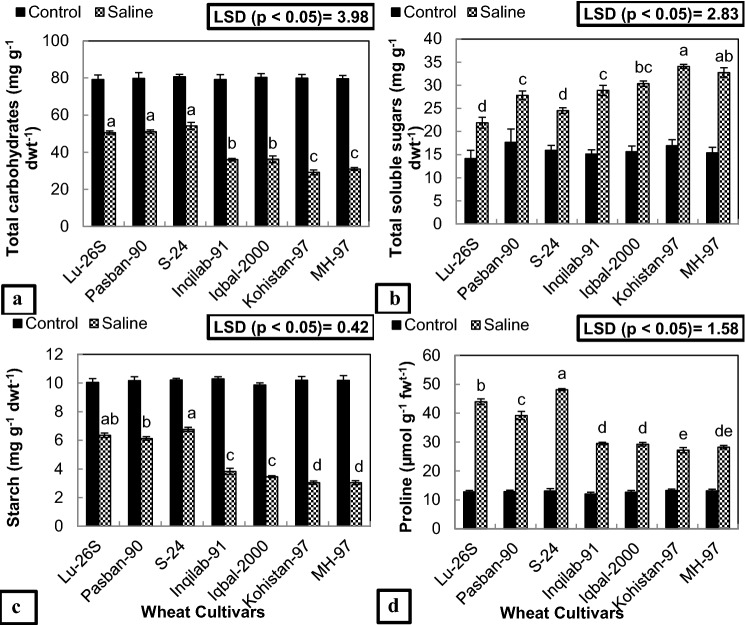
Concentration of organic osmolytes of seven selected salt tolerant and sensitive wheat cultivars subjected to 0 or 150 mM NaCl stress at adult stage. Different letters represent significant differences among the cultivars at (*p* < 0.05) by LSD test. (**a**) Total carbohydrates, (**b**) Total soluble sugars, (**c**) Starch and (**d**) Proline.

ANOVA of the data for total soluble sugars and proline concentration show significant (*P* ≤ 0.001) increase while for total carbohydrates and starch show significant (*P* ≤ 0.001) decrease in all the cultivars due to imposition of salt stress (Table 2). About 54–63% decrease in total carbohydrates was observed in sensitive cultivars (Kohistan-97, MH-97, Inqilab-91 and Iqbal-2000), while less decrease ~ 32–36% was calculated in salt tolerant (S-24, LU-26S and Pasban-90) cultivars due to imposition of salt in the growth medium (Fig. 2a). Application of 150 mM NaCl in growth medium caused 53% increase in total soluble sugar content of S-24, 55% in LU-26S and 57% in Pasban-90, while the cv. Kohistan-97 showed maximum increase (113%) in concentration of total soluble sugar content, the second was MH-97 with 101% increase and cvs. Iqbal-2000 and Inqilab-91 are placed at the end with 93% and 90% increase respectively in total soluble sugar content under the influence of NaCl stress (Fig. 2b). Likewise, starch content of leaves decreased in all wheat varieties due to salt stress. However, such reduction in starch content was lower (~ 33–39%) in salt tolerant varieties as compared to (~ 62–70%) salt-sensitive wheat varieties (Fig. 2c). Proline achieved its highest level in S-24 (48 µmol g^−1 ^fwt^−1^) after that (~ 39–43 µmol g^−1 ^fwt^−1^) in LU-26S and Pasban-90 whereas minimum concentration (27 µmol g^−1 ^fwt^−1^) was found in Kohistan-97, while MH-97, Iqbal-2000 and Inqilab-91 are next to them (~ 28–29 µmol g^−1 ^fwt^−1^) under salinity (Fig. 2d). Comparison of means showing different letters by LSD test at *p* < 0.05 represent significant differences among the cultivars for these organic osmolytes.

**Table 2:** Mean squares from ANOVA of the data for concentration of anti-oxidant enzymes, MDA, organic osmolytes and total chlorophyll content of seven selected wheat cultivars subjected to 150 mM NaCl salinity.

Source of variation	df	CAT	POD	APX	SOD	MDA
Cultivars	6	0.524***	0.875***	32.41***	6361.75*	3.828***
Salinity	1	25.71***	48.96***	2685.1***	190,258.4***	397.03***
Cultivars x Salinity	6	0.48***	0.773***	39.57***	11,342.02***	5.023***
Error	56	0.06	0.047	2.590	2374.94	1.388
Total	69					

Source of variation	df	Total carbohydrates	Total soluble sugars	Starch	Proline	Total chlorophyll content
Cultivars	6	280.8***	61.23***	7.09***	185.72***	17.85***
Salinity	1	26,192.5***	2860***	524.93***	8651.31***	2117.5***
Cultivars x Salinity	6	269.4***	39.2***	7.02***	181.47***	26.29***
Error	56	9.873	5	0.11	1.56	4.425
Total	69					

*df* degree of freedom, *CAT* catalase, *POD* peroxidase, *APX* ascorbate peroxidase, *SOD* superoxide dismutase, *MDA* Malondialdehyde.

*, **, *** significant at 0.05, 0.01, and 0.001 probability.

**Figure 3:**
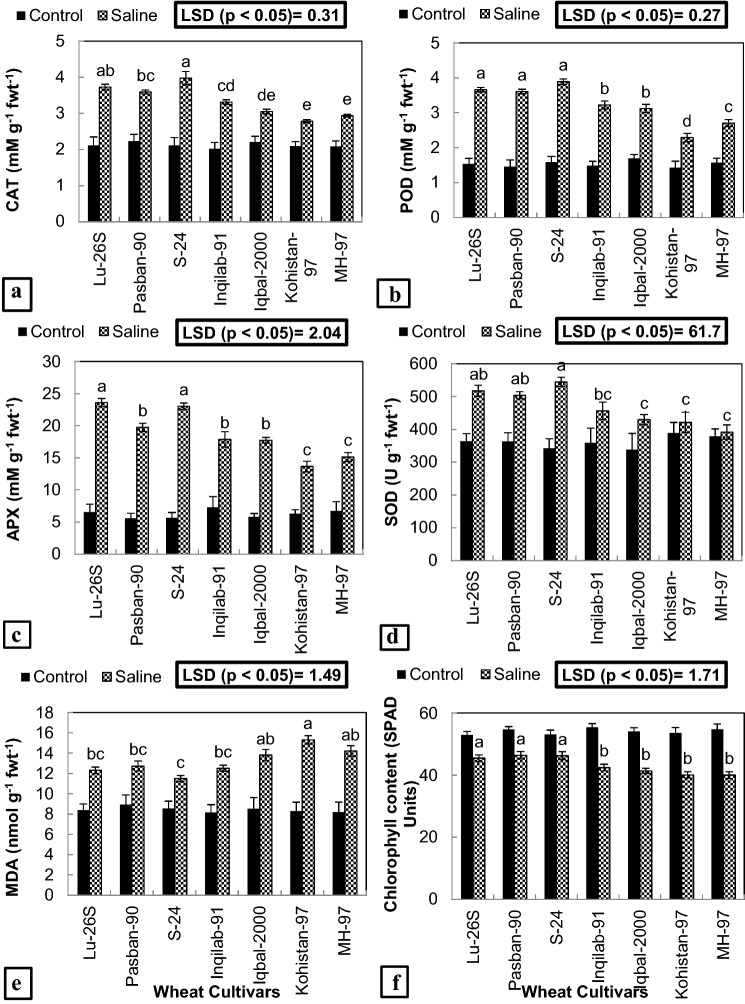
Concentration of antioxidant enzymes, MDA and chlorophyll content (SPAD values) of seven selected salt tolerant and sensitive wheat cultivars subjected to 0 or 150 mM NaCl stress at adult stage. Different letters represent significant differences among the cultivars at (*p* < 0.05) by LSD test. CAT = Catalase, POD = Peroxidase, APX = Ascorbate peroxidase, SOD = Superoxide dismutase, MDA = Malondialdehyde. (**a**) CAT activity, (**b**) POD activity, (**c**) APX activity, (**d**) SOD activity, (**e**) MDA content and (**f**) Chlorophyll content.

Anti-oxidants such as CAT, POD, APX and SOD as well as MDA concentration significantly (*P* ≤ 0.001) increased while chlorophyll content significantly (*P* ≤ 0.001) decreased in all the cultivars (Fig. 3, Table 2) under salt stress. Maximum increase in concentration of CAT, APX and SOD was observed in S-24 followed by LU-26S and Pasban-90 respectively due to salt stress (Fig. 3a, c, d) while for POD, Pasban-90 displayed maximum increase under the influence of salt stress and S-24 and LU-26S followed (Fig. 3b). MH-97 and Kohistan-97 showed minimum increase in the concentration of these enzymes while Iqbal-2000 and Inqilab-91 exhibited intermediate increase in the concentration of antioxidant enzymes under saline conditions (Fig. 3a–d). For MDA concentration, only 34% increase in S-24, 46% in LU-26S and 53% in Pasban-90 was observed under salinity stress, however maximum increase (84%) was found in Kohistan-97 followed by 73% in MH-97 while Iqbal-2000 and Inqilab-91 showed intermediate increase in amount of MDA in salt treated plants (Fig. 3e). Chlorophyll content (SPAD index) show minimum decrease in varieties S-24, LU-26S and Pasban-90 while maximum in MH-97 followed by Kohistan-97, Iqbal-2000 and Inqilab-9 (Fig. 3f). Comparing means by LSD test at *p* < 0.05 for these parameters represent significant differences among the cultivars with different letters.

Data obtained from adult experimental parameters recommended S-24 and LU-26S as the most salt tolerant while MH-97 and Kohistan-97 as the most salt sensitive cultivars among the seven selected cultivars from screening experiment.

### Chlorophyll a fluorescence transient

**Figure 4:**
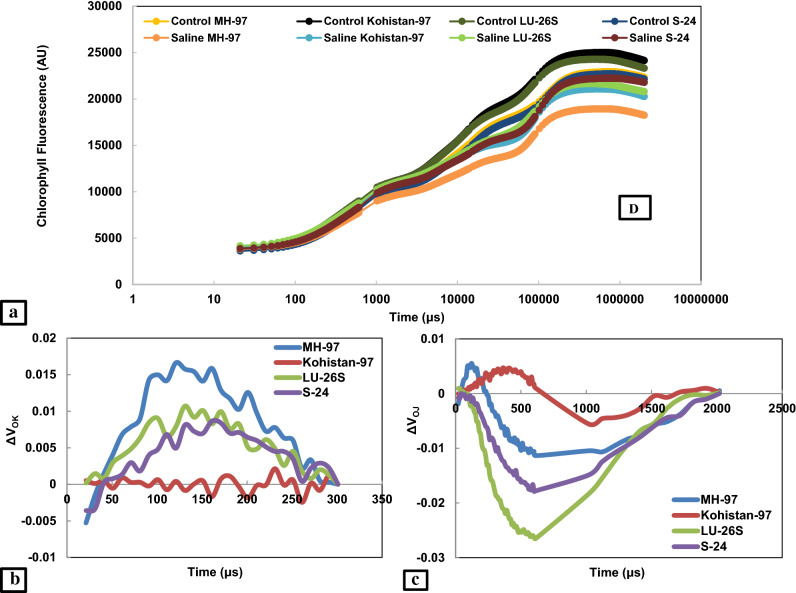
Kinetic differences of four selected wheat cultivars subjected to 0 or 150 mM NaCl stress (**a**) Raw OJIP chlorophyll a fluorescence transients (**b**) Double normalized between Fo and Fk (L-band) (**c**) Double normalized between Fo and Fj (K-band).

**Figure 5:**
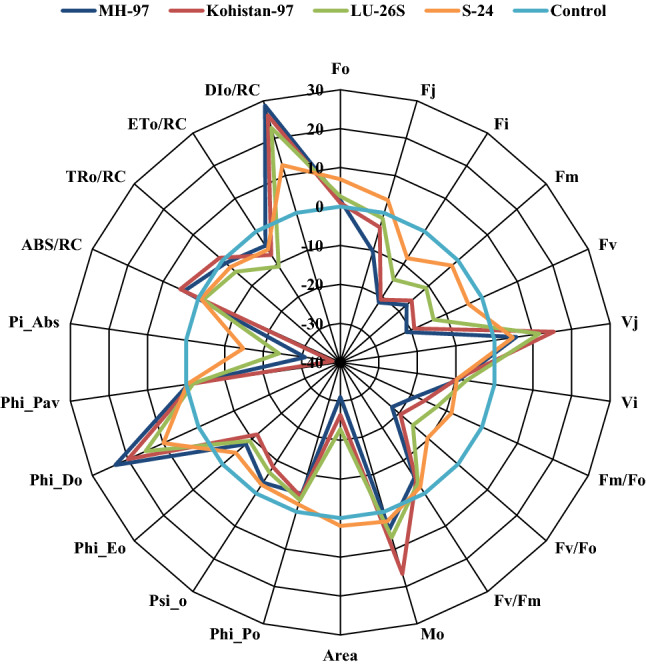
Radar plot of selected JIP test parameters showing changes in chlorophyll a fluorescence transients (normalized to 0 mM NaCl as reference) of four selected wheat cultivars subjected to 150 mM NaCl stress at vegetative stage.

**Figure 6:**
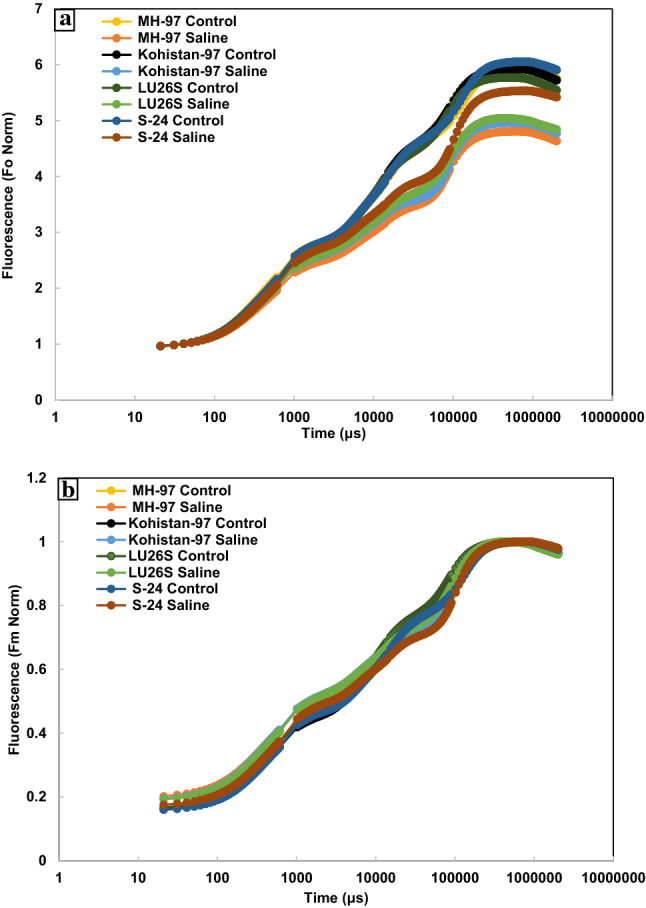
Normalized OJIP chlorophyll *a* fluorescence transients of (**a**) Fo and (**b**) Fm for four wheat cultivars subjected to 0 or 150 mM NaCl stress at vegetative stage.

**Figure 7:**
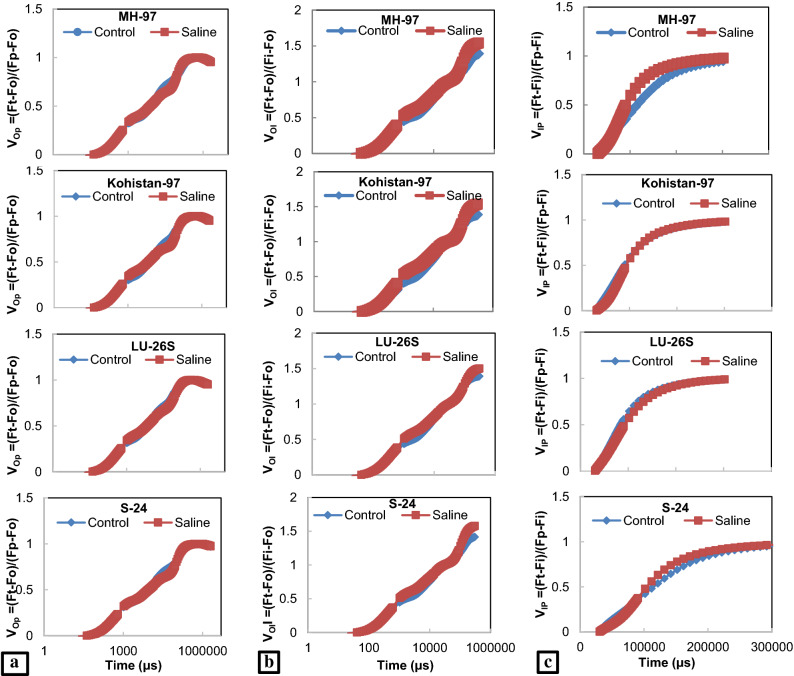
OJIP chlorophyll *a* fluorescence transients double normalized between (**a**) Fo and Fm (V_OP_) (**b**) Fo and Fi (V_OI_) and (**C**) Fi and Fp (V_IP_) of four wheat cultivars subjected to 0 or 150 mM NaCl stress at vegetative stage.

In present study, JIP test was used to analyse chlorophyll *a* fluorescence transient which express the damage caused by salt stress to photosynthetic machinery. Chlorophyll *a* fluorescence transients explain that fluorescence emission remained low at Fj, Fi and Fm steps of OJIP curve except for Fo where small increase was observed in all cultivars under salt stress. Open PSII reaction centres and oxidized state of QA was expressed by Fo while closed PSII reaction centres and reduced state of QA as Fm. Extant of decrease in chlorophyll fluorescence under salt stress was more evident in MH-97 and Kohistan-97 especially at J, I and P steps while minimum in S-24 and LU-26S as clear from Fig. 4a and radar plot (Fig. 5). Salt stress caused damage to OEC reducing Fv values which is an indication of lowering PSII ability to reduce plastoquinone. Greater decrease was observed in MH-97 and Kohistan-97 as compared to cvs. S-24 and LU-26S (Fig. 5). Salinity reduces transfer of electrons from QA to electron transport chain causing decrease in plastoquinone pool (PQH_2_) and greater increase in dissipation of light energy (DIo/RC) in MH-97 and Kohistan-97 compared to S-24 and LU-26S, so PI_ABS_ is reduced more in MH-97 and Kohistan-97 while less reduction in S-24 and LU-26S was observed (Fig. 5). Maximum PSII quantum yield (Fv/Fm ratio or Φ Po), electron transport rate through PSII (Fm/Fo) and quantum efficiency of PSII (Fv/Fo) were significantly reduced at 150 mM NaCl stress and the decrease was more prominent in MH-97 and Kohistan-97 compared to S-24 and LU-26S genotypes. Energy fluxes (including ABS/RC, TRo/RC, ETo/RC and DIo/RC) are significantly influenced by salinity stress in contrasting wheat genotypes, but are greatly affected in sensitive genotypes compared to tolerant (Fig. 5). Chlorophyll *a* fluorescence transients normalized with Fo and Fm also indicate maximum decrease in MH-97 and Kohistan-97 compared to S-24 and LU-26S (Fig. 6). Decreased fluorescence shows poor electron transport efficiency of PSII in MH-97 and Kohistan-97 rendering them vulnerable to salinity. Normalized transient curves of control and salt treated plants were expressed as OP, OJ and OK. Chlorophyll *a* fluorescence transient double normalized between steps O (Fo) and P (F_P_) is represented by V_OP_. Under control conditions, the increase in V_OP_ fluorescence from 0.1 to 1 ms was slower but application of salinity treatment made it faster as explained in Fig. 7a. Kinetic differences between double normalized salt stressed and control plants for chlorophyll *a* fluorescence values over a time range of 20 to 300 μs (between O and K steps) is called ∆V_OK_ or L-band (Fig. 4b) while the values over a time range of 20–2000 μs (between O and J steps) as ∆V_OJ_ or K-band (Fig. 4c). L-band expresses PSII subunits connectivity while K-band represents OEC activity. Low fluorescence at 1000 μs and then increasing peaks from 1000 to 2000 μs in K-band expressed reduced OEC performance due to disturbance in electron flow from OEC to PSII reaction centre. K-band values for S-24 and LU-26S show that plants maintain antenna complex and PSII reaction centre which make them tolerant to salinity while their disruption make MH-97 and Kohistan-97 as sensitive cultivars (Fig. 4c). L-band expresses uncoupling of OEC, its increased value in MH-97 make it most sensitive as compared to others as shown in Fig. 4b. OJIP chlorophyll *a* fluorescence transient at IP phase can be evaluated by two ways: (1) V_OI_ shows variable fluorescence transients double normalized between F_O_ and F_I_ phases. Kinetic properties for oxidation/reduction of PQ pool are represented by OI part of the curve, negative peak under salt stress expresses ability to maintain PQ reduction rate (7b). (2) V_IP_ expresses variable fluorescence transients double normalized between F_I_ and F_P_ phases. V_IP_ was increased for four wheat cultivars due to salinity, maximum in MH-97 (sensitive) and minimum in LU-26S (tolerant) (Fig. 7c) due to reduced rate of electron flow from intersystem to PSI possibly due to damage in reaction centre of PSII.

## Discussion

Considerable efforts have been made to develop salt tolerant wheat cultivars, but a little success has been achieved in this regard due to lack of detailed knowledge on any workable salt tolerance strategy, variation in mechanism of salt tolerance in different wheat cultivars, poor selection techniques and non-availability of novel sources of salt tolerance ^11,27,28^.

Germplasm of 40 wheat lines was screened for salt tolerance at early growth, vegetative and adult stages and finally two salt tolerant (S-24 and LU-26S) and two sensitive (MH-97 and Kohistan-97) wheat lines/cultivars were identified against salt stress. The cultivars S-24, LU-26S and Pasban-90 (salt tolerant) exhibited less decrease in shoot and root fresh and dry biomass, P and K^+^ contents and maximum exclusion of Na^+^ in the respective order at seedling stage as compared to MH-97, Kohistan-97, Inqilab-91 and Iqbal-2000 (salt sensitive cultivars) under saline conditions. Growth related parameters are important screening tools to assess salt tolerance in plants because metabolites are consumed towards protection against salt induced damage instead of growth, which is reduced^2^. Low Na^+^ contents in leaves of salt tolerant cultivars show their ability to exclude Na^+^ ions from their shoots^29^. Sofy, et al.^30^ observed decline in N, P and K^+^ contents while increased accumulation of Na^+^ due to salt treatment in common bean (*Phaseolus vulgaris* L.) while in ridge gourd (*Luffa acutangula* L.) by Shahzadi, et al.^31^. In present study reduced Na^+^ translocation to leaves is observed in tolerant genotypes to protect photosynthetic machinery from toxic effects of high concentration of NaCl.

Compatible solute accumulation for osmotic adjustment such as proline, sugar, starch and carbohydrates is an important tolerance mechanism in plants against stresses^32^. Greater concentration of proline plays important role in osmotic adjustment, ROS scavenging and stabilizing cellular components^33^. Greater rise in proline concentration and soluble sugars in S-24, LU-26S and Pasban-90 under salinity make them salt tolerant while less accumulation of these osmolytes confer salt sensitivity to MH-97, Kohistan-97, Inqilab-91 and Iqbal-2000 in respective order. S-24 was considered to be the most salt tolerant and LU-26S was next to it while Kohistan-97 was considered to be the most salt sensitive and MH-97 followed (Fig. 2a–d). In previous studies higher accumulation of total soluble sugars in wheat genotypes under salinity treatment was observed by Hussain, et al.^34^. Increase in proline concentration due to salt treatment has been reported in wheat and rice^33^.

ROS such as superoxide (O_2_^•–^), H_2_O_2_, hydroxyl radical (OH^•^) and singlet oxygen (^1^O_2_) produced under high salinity in wheat cause membrane damage in various cytoplasmic organelles producing MDA^35^. Lesser amount of MDA found during present study in tolerant cultivars (S-24, LU-26S and Pasban-90) represents low membrane lipid peroxidation and more protection against oxidative damage as compared to salt sensitive cultivars (Kohistan-97, MH-97, Inqilab-91 and Iqbal-2000) (Fig. 3e). Previous studies have shown increased ROS production causing membrane damage in salt treated plants of tomato, rice, pea, citrus and wheat^36^. To mitigate the effect of oxidative stress under saline conditions, activation of genes expressing anti-oxidative enzymes such as APX, SOD, CAT and POD occurs in plants as explained by Ahanger, et al.^37^. Higher concentration of anti-oxidative enzymes in tolerant cultivars has been found under salinity stress possibly in response of damage caused by ROS to photosynthetic apparatus^30^. Higher amount of anti-oxidant enzymes observed in recent study makes S-24, LU-26S and Pasban-90 as salt tolerant cultivars while their lesser concentration suggests Kohistan-97, MH-97, Inqilab-91 and Iqbal-2000 as salt sensitive varieties (Fig. 3a–d). Previous studies also explain higher activities of POD, CAT, SOD and APX and less MDA contents in salt tolerant varieties of wheat and sesame^29,38^.

Effect of salt stress on photosynthesis in plants can be explained by chlorophyll *a* fluorescence parameters which describe damage to donor or acceptor side of PSII, inhibition of electron transport and changes in oxidation and reduction in QA, QB and PQ^10^. Increasing Fo, in recent study suggests decreased energy trapping capacity of PSII while the decrease in Fm values may be attributed to inactive reaction centres under the influence of salt stress. Donor side of PSII (OEC) is very sensitive to salt stress and its damage cause decline in Fv as a result PSII capacity to reduce plastoquinone is adversely affected^39^. PSI and PSII oxidation is inhibited under reduced Fv/Fo (quantum efficiency of PSII) resulted from reduced electron transport rate (Fm/Fo) through PSII or photo-damage caused by salinity to photosynthetic apparatus^40^.

In present study, negative K-band values of tolerant cultivars (Fig. 4c) is in accordance with the previous studies which explain that K-step gives information about the destruction of OEC^41^, while L-band expresses energetic connectivity of antenna to PSII reaction centre units^42^. Electron transport efficiency of PSII remains poor due to imposition of salt stress in wheat plants which reduces light harvesting potential of PSII by lowering ABS/RC due to reduction in photosynthetic pigments and increase in inactive reaction centres reducing ETo/RC (Fig. 5). Previously, decreased values of ABS/RC, ETo/RC and TRo/RC in salt treated oat plants were observed by Varghese, et al.^43^. Reduction in Ψo indicates blockage of electron transfer from reduced QA to QB which may be due to disruption of thylakoid membrane of photosynthetic pigments as explained by Kalaji, et al.^44^. Results of present study also show reduction in transfer of electron from QA to electron transport chain (Ψo) decreasing plastoquinone pool (PQH_2_) and increasing light energy dissipation (DIo/RC), ultimately PI_ABS_ is reduced (Fig. 5). Maximum yield of primary photochemistry (ΦPo) also called Fv/Fm ratio is reduced under salinity because of damage in PSII reaction centre. In present experiment more decrease of Fv/Fm in sensitive genotypes (MH-97 and Kohistan-97) compared to tolerant (S-24 and LU-26S) was observed under salinity (Fig. 5) which is in agreement with previous studies by Kalaji, et al.^45^ where salt stressed barley plants have only 25% Fv/Fm values of control plants due to enhanced photochemical inactive reaction centres. Sun, et al.^46^ reported that decreased efficiency of light reaction results in increased energy dissipation as heat ultimately efficiency of reaction centres is depressed. Reduction in quantum yield of PSII in wheat under salinity has been recently explained by Hussain, et al.^34^. Arabidopsis plants exhibited 76% reduction in quantum efficiency of PSII due to two weeks of salt treatment^43,47^. PI_ABS_ depends upon the concentration of active reaction centres of chlorophyll, efficiency of energy trapping and electron transport from PSII to the plastoquinone pool^48^. PSII Fv/Fm and PI_ABS_ are important screening tools to investigate photosynthetic efficiency and effects of abiotic stresses on PSII in different plants^49,50^. Rate of energy dissipation by PSII (DIo/RC) and maximum quantum yield of non-photochemical de-excitation (ΦDo), both increase under salt stress which indicate dissipation of absorbed light as heat and confer tolerance against salinity as observed in present investigation and also explained in previous studies by Varghese, et al.^43^. In recent study the maximum quantum yield of electron transport (ΦEo) was reduced under salt stress, greater decrease was observed in sensitive cultivars (MH-97 and Kohistan-97) compared to tolerant cultivars (S-24 and LU-26S) (Fig. 5), similar results were reported earlier in rice seedlings and *Brassica napus* L.^49^ (Supplementary Table S1).

## Conclusion

Two wheat cultivars S-24 and LU-26S had greater salt tolerance than the rest of local wheat germplasm including previously known salt tolerant cv. Pasban-90. Differential metabolic and physiological responses of contrasting wheat genotypes were found. Among them, most important attributes were higher K^+^ uptake over Na^+^, greater accumulation of soluble sugars and proline, higher activity of antioxidant enzymes and structural stability of photosystem-II. Measurements of chlorophyll *a* fluorescence transients suggested that better efficiency of photosystem II in tolerant cultivars (S-24 and LU-26S) was due to better management of absorbed energy (ABS/RC) by down-regulating electron transport per reaction centre (ETo/RC) and enhanced energy dissipation (DIo/RC) to prevent photosystem-II from photo oxidative damage. Greater PSII stability and functional activity in salt tolerant cultivars S-24 and LU-26S was evident with greater performance index (PI_ABS_) and greater quantum yield (Fv/Fm) of PSII (Supplementary Fig. S1).

## Supplementary Information

Supplementary Figures.

Supplementary Table 1.

## Data Availability

The datasets used and/or analysed during the current study are available from the corresponding author on reasonable request.
