# Supplementary material for: Role of mineral nutrients, antioxidants, osmotic adjustment and PSII stability in salt tolerance of contrasting wheat genotypes
Source: Sci Rep. 2022 Jul 25;12:12677. doi: 10.1038/s41598-022-16922-9 (PMC9314327; doi:10.1038/s41598-022-16922-9)
Supplement: Supplementary file 1 — Supplementary Figures. [file 41598_2022_16922_MOESM1_ESM.pdf]

Role of mineral nutrients, antioxidants, osmotic adjustment and PSII stability in exploring salt tolerance potential in contrasting wheat genotypes

Nadeem Hussain[1,*], Younas Sohail[2], Nasir Shakeel[3,*], Muhammad Javed[4], Hussan Bano[5], Hafiza Saima Gul[1], Zafar Ullah Zafar[1], Islam Frahat Zaky Hassan[6], Abdul Ghaffar[1], Habib-ur-Rehman Athar[1], Rahaf Ajaj[7]

[1] Institute of Pure and Applied Biology, Bahauddin Zakariya University, Multan 60800, Pakistan
[2] Department of Botany, Emerson University, Multan, Pakistan
[3] Department of Inorganic, Analytical Chemistry and Electrochemistry, Faculty of Chemistry, Silesian University of Technology, Gliwice, Poland
[4] Department of Botany, Division of Science and Technology, University of Education, Lahore, Pakistan
[5] Department of Botany, The Women University, Multan, Pakistan
[6] National Research Center, Agricultural and Biology Research Institute, Water Relations and Field irrigation Department, Cairo, Egypt
[7] College of Health Sciences, Abu Dhabi University, UAE
Authors for correspondence:
*e-mail: nasir.shakeel@polsl.pl
*e-mail: nadeemhussain396@yahoo.com

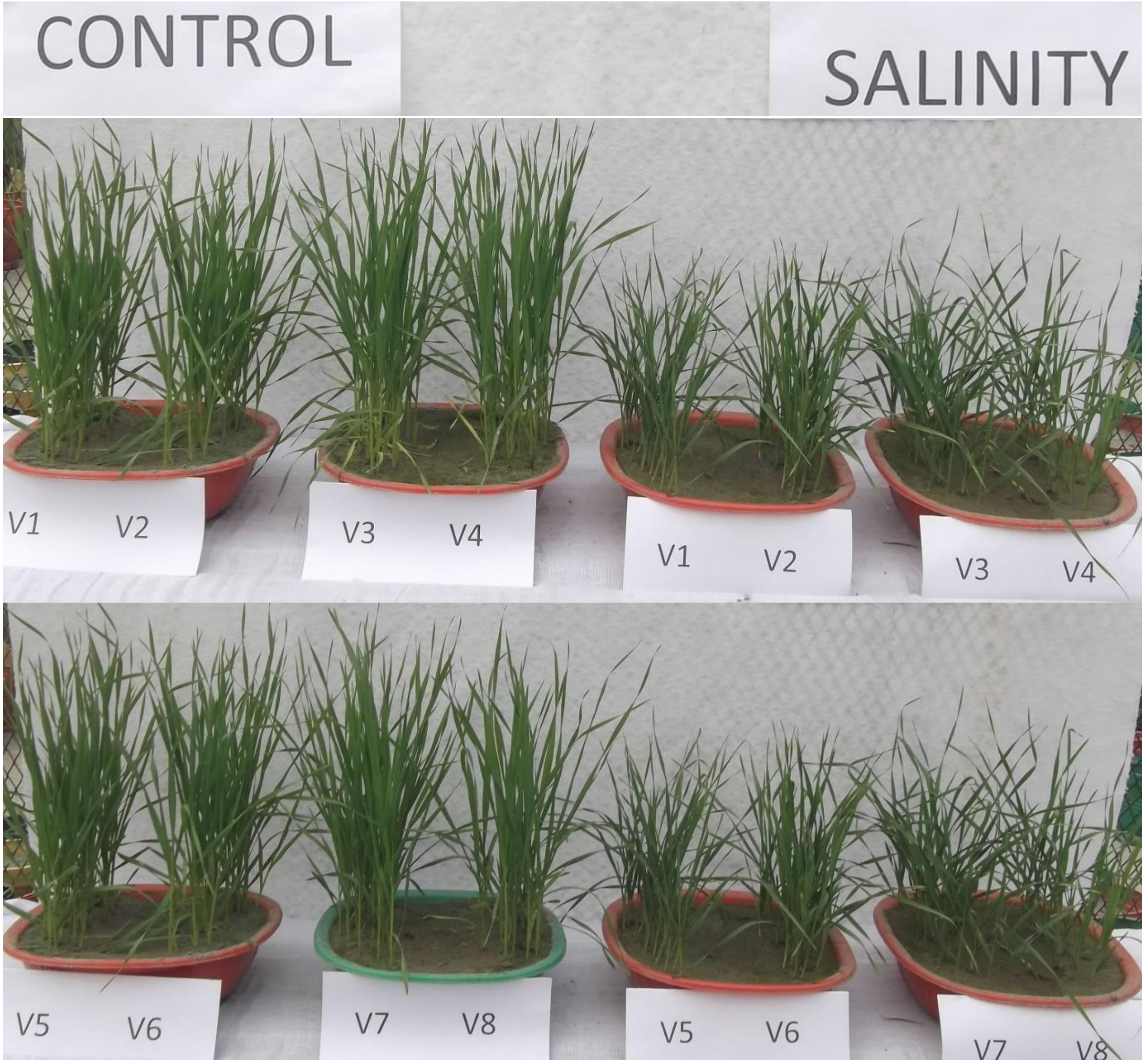

V1. AARI -11, V2. AAS-11, V3. AAS-2000, V4. Barani-83, V5. BARS-09, V6. Blue Silver, V7. Chenab-70, V8. Chenab-2000

Supplementary Figure S1. Plants of local wheat genotypes (V1-V8) at seedling stage grown under control (0 mM) or 150 mM NaCl salinity stress.

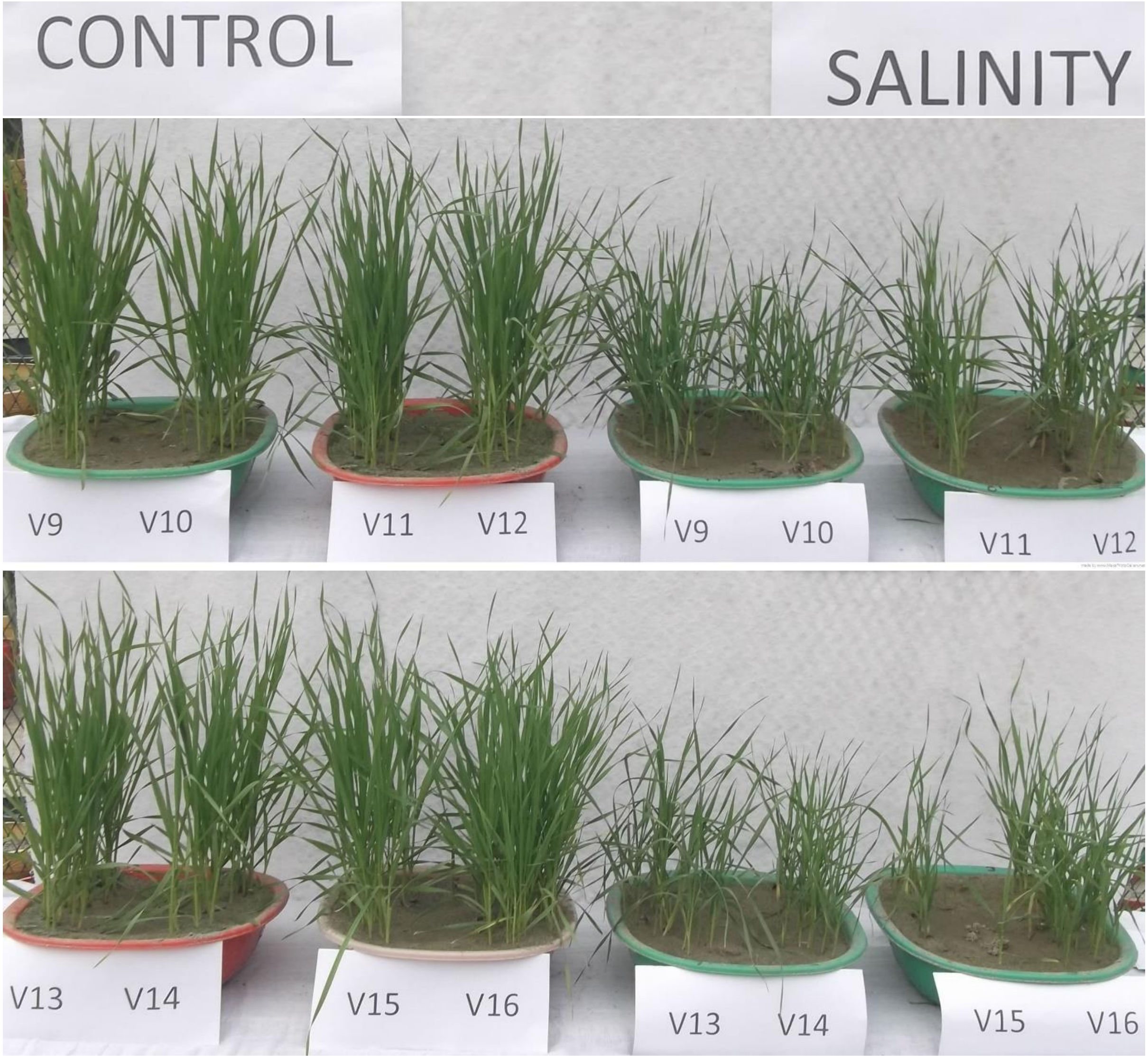

V9. D-97, V10. FSD-08, V11. FSD-83, V12. FSD-85, V13. Galaxy-13, V14. Inqilab-91, V15. Iqbal-2000, V16. Kohinoor-83

Supplementary Figure S2. Plants of local wheat genotypes (V9-V16) at seedling stage grown under control (0 mM) or 150 mM NaCl salinity stress.

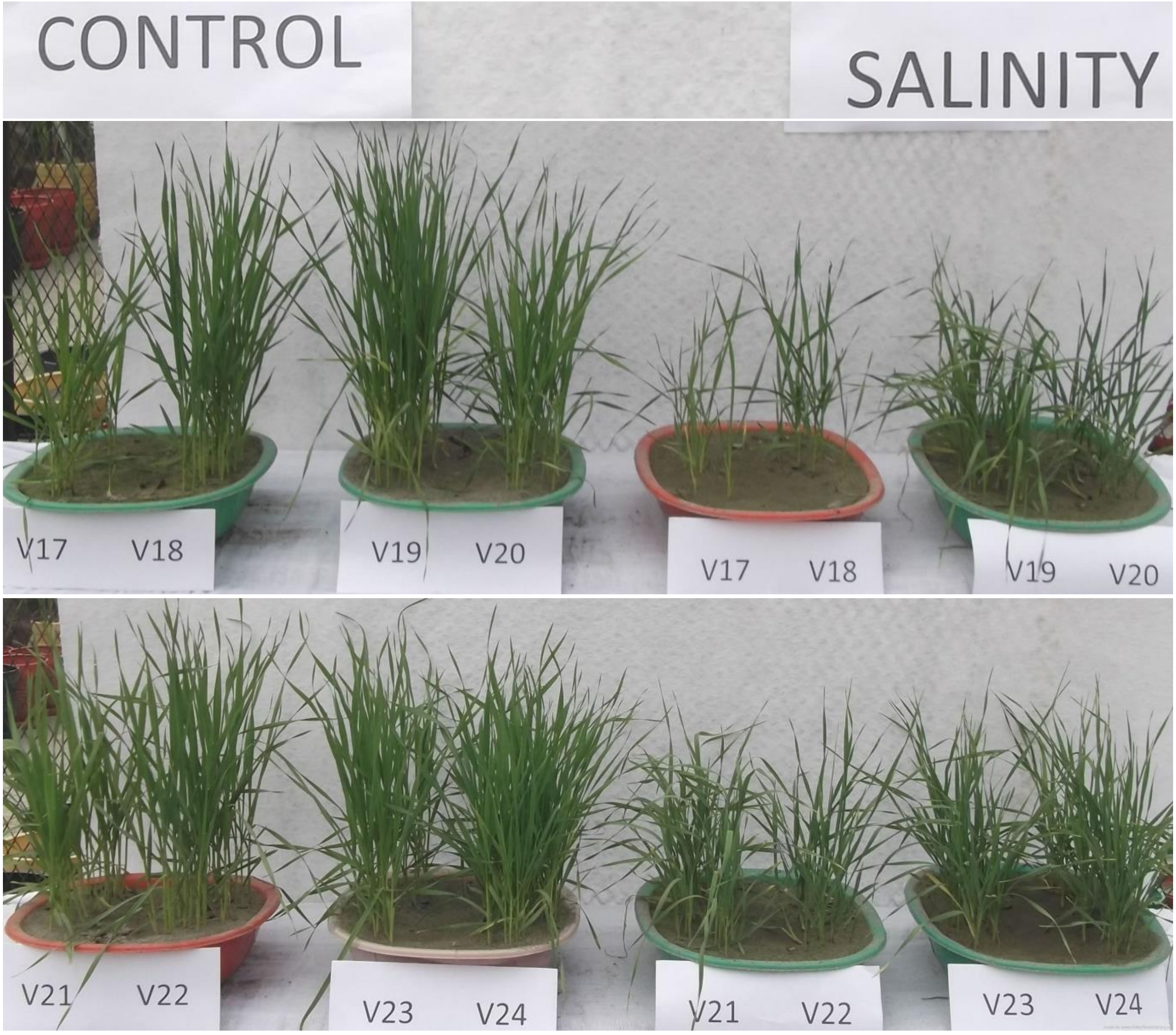

V17. Kohistan-97, V18. Lasani-08, V19. LU-26S, V20. Lyp-73, V21. Millat-11, V22. M.Pak-65, V23. M.H-97, V24. NARC-11

Supplementary Figure S3. Plants of local wheat genotypes (V17-V24) at seedling stage grown under control (0 mM) or 150 mM NaCl salinity stress.

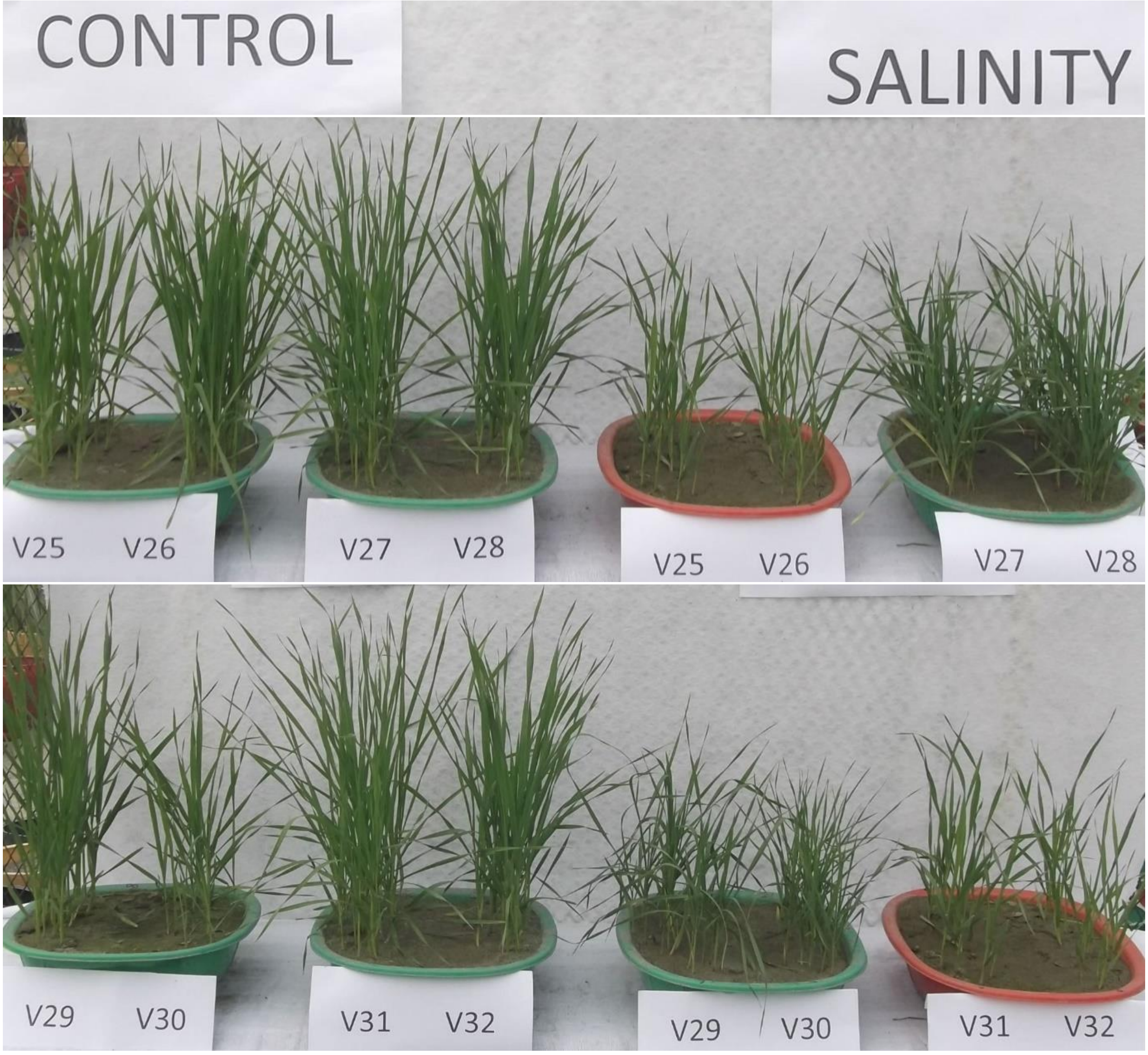

V25. Pak-13, V26. Pak-81, V27. Parwaz-94, V28. Pasban-90, V29. Punjab-11, V30. Punjab-76, V31. Punjab-85, V32. Punjab-96

Supplementary Figure S4. Plants of local wheat genotypes (V25-V32) at seedling stage grown under control (0 mM) or 150 mM NaCl salinity stress.

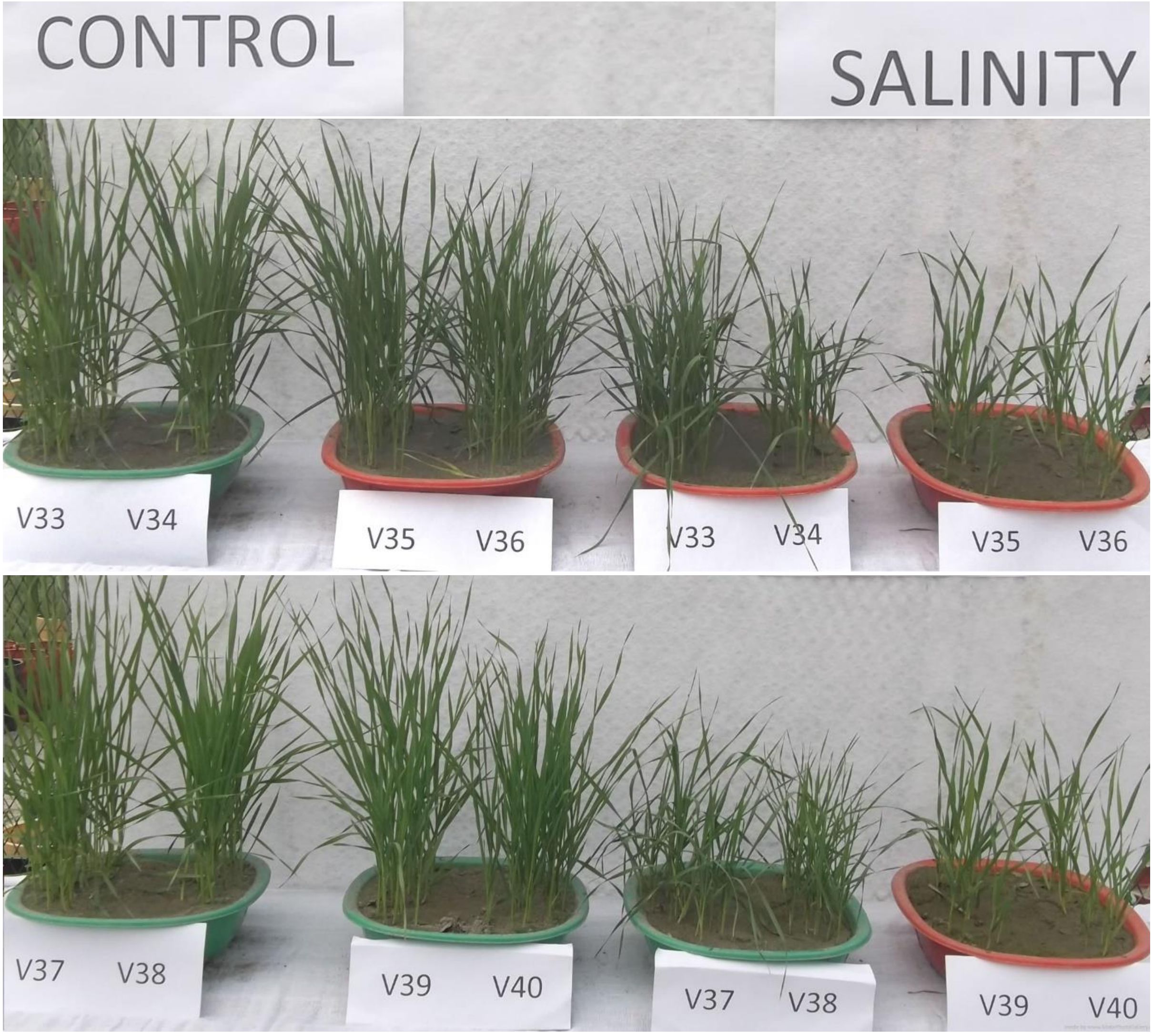

V33. S-24, V34. SH-02, V35. Shahkar-95, V36. S.A-75, V37. Sahar-06, V38. Shafaq-06, V39. Uqaab-2000, V40. WL-711

Supplementary Figure S5. Plants of local wheat genotypes (V33-V40) at seedling stage grown under control (0 mM) or 150 mM NaCl salinity stress.
