# Supplementary material for: Role of mineral nutrients, antioxidants, osmotic adjustment and PSII stability in salt tolerance of contrasting wheat genotypes
Source: Sci Rep. 2022 Jul 25;12:12677. doi: 10.1038/s41598-022-16922-9 (PMC9314327; doi:10.1038/s41598-022-16922-9)
Supplement: Supplementary file 2 — Supplementary Table 1. [file 41598_2022_16922_MOESM2_ESM.pdf]

Supplementary Table S1. The origin and pedigree of selected salt tolerant and sensitive wheat genotypes

Year	Variety	Breeder/origin	Pedigree
1978	LU-26S	UAF	BLS-KHUSHAL 69<br>**Shahid Mukhtar, et al.** [1]
1994	S-24	BZU, Multan	LU- 26S/Kharchia<br>**Ashraf** [2]
1990	PASBAN-90	AARI, Faisalabad	INIA66/A.DISST//INIA66/3/GEN81<br>**Shahid Mukhtar, et al.** [1]
1991	INQILAB-91	AARI, Faisalabad	WL711/CROW<br>Shahid Mukhtar, et al. [1]
2000	IQBAL-2000	AARI, Faisalabad	BURGUS/SORT-12-13 //KAL/BB /3/PAK81<br>**Shahid Mukhtar, et al.** [1]
1997	KOHISTAN-97	AARI, Faisalabad	V-1562//CHRC/HORK/3KUFRA-1/4/CARP/BJY<br>**Shahid Mukhtar, et al.** [1]
1997	MH-97	AARI, Faisalabad	ND/VG69144/KOL/BB/3 YACO/4 VEE # 5<br>**Shahid Mukhtar, et al.** [1]

UAF = University of Agriculture, Faisalabad, BZU = Bahauddin Zakariya University

AARI = Ayyub Agricultural Research Institute

1 Shahid Mukhtar, M., Rahmanw, M.-u. & Zafar, Y. Assessment of genetic diversity among wheat (Triticum aestivum L.) cultivars from a range of localities across Pakistan using random amplified polymorphic DNA (RAPD) analysis. *Euphytica* **128**, 417-425 (2002).

2 Ashraf, M. Registration of ‘S-24’Spring Wheat with Improved Salt Tolerance. *Journal of plant registrations* **4**, 34-37 (2010).
